# Automated Tractography Settings for Assessing the Corticoreticular Pathway in Patients With Stroke: A Clinical Application

**DOI:** 10.7759/cureus.111590

**Published:** 2026-06-26

**Authors:** Tetsuo Koyama, Midori Mochizuki, Yuki Uchiyama, Kazuhisa Domen

**Affiliations:** 1 Department of Rehabilitation Medicine, Nishinomiya Kyoritsu Neurosurgical Hospital, Nishinomiya, JPN; 2 Department of Rehabilitation Medicine, School of Medicine, Hyogo Medical University, Nishinomiya, JPN

**Keywords:** automated tractography, corticoreticular pathway, japanese rehabilitation, neuroimaging, outcome, prognosis, recovery, stroke, tract, tractography

## Abstract

Background: The corticoreticular pathway (CRP) is gaining attention for its potential role in post-stroke motor recovery. However, unlike the corticospinal tract (CST), the CRP is not included in commonly used automated tractography frameworks, limiting its reproducibility and clinical applicability. We aimed to incorporate CRP assessment into an automated tractography pipeline and to evaluate its feasibility in patients with mild stroke.

Methods: We retrospectively analyzed patients with a first-ever unilateral supratentorial ischemic stroke who had regained full independence in activities of daily living. Diffusion tensor imaging was acquired during the second week after admission using a 3.0-T MRI scanner. Tractography was performed using a fully automated procedure that reconstructed both the CST and the CRP. For CRP reconstruction, the seed region was placed in the gigantocellular reticular nucleus and the target region in the premotor cortex, based on established neuroanatomical atlases. Tract volume, fractional anisotropy (FA), and CST-CRP overlap were quantified. Lesioned and non-lesioned hemispheres were compared using the Wilcoxon signed-rank test.

Results: Fifteen patients met these criteria during the study period. Automated tractography consistently reconstructed both pathways in all participants. CRP volume tended to exceed CST volume, although with substantial interindividual variability. FA values were relatively preserved in the lesioned hemisphere but were significantly lower than in the non-lesioned hemisphere for both the CST (S = 48,P = 0.004) and the CRP (S= 54, P = 0.001).

Conclusions: Using anatomically defined regions of interest enabled reliable automated CRP reconstruction. This approach offers a practical and reproducible method for evaluating motor-related white matter pathways and may facilitate future research into stroke rehabilitation.

## Introduction

Neuroimaging plays a critical role in stroke rehabilitation by providing objective insights into neural damage and recovery potential [1]. Among the available imaging modalities, diffusion tensor imaging (DTI) is particularly valuable because it enables the in vivo assessment of the microstructural integrity of white matter pathways [1].

Traditionally, the corticospinal tract (CST) has been considered the principal neural substrate governing the motor control of both upper and lower extremities [2]. More recently, growing attention has been directed toward the corticoreticular pathway (CRP) due to its potential contribution to motor recovery in affected extremities. Unlike the CST, which serves as the primary pathway for voluntary movement, the CRP is increasingly recognized for its role in modulating motor control and compensating for CST damage [3-7].

Among various DTI-based analytical techniques, tractography provides three-dimensional tract reconstructions of major white matter pathways [8]. This visualization is clinically relevant as it allows both clinicians and patients to intuitively understand stroke-related neural pathology [9]. However, conventional tractography requires the manual definition of seed and target regions, making the process time-consuming, operator-dependent, and susceptible to low reproducibility [10]. To overcome these limitations, the automated and standardized tractography framework XTRACT was recently developed [11]. Although it routinely reconstructs 42 major tracts, including the CST, the CRP is not included in its original protocol.

To enhance the clinical applicability of automated tractography techniques in stroke rehabilitation, we extended this framework to incorporate CRP reconstruction based on established anatomical atlases. This technical report details the methodological refinement, motivated by the increasing recognition of the CRP as a potential substrate for motor recovery in affected extremities.

## Materials and methods

Patients

A retrospective cohort analysis was conducted based on a clinical record review. Eligible participants were patients admitted to Nishinomiya Kyoritsu Neurosurgical Hospital for stroke treatment between April 2022 and March 2024. To minimize heterogeneity related to premorbid functional status and lesion characteristics, we included only individuals with a first-ever unilateral supratentorial ischemic stroke who had been independent in activities of daily living prior to stroke onset [12]. Eligibility criteria prohibited the inclusion of patients with pre-existing neurological conditions like Alzheimer’s or Parkinson’s disease. Similarly, the study population did not include individuals who developed impaired consciousness during the course of the study or those with severe concurrent medical illnesses.

The primary aim of the study was to establish an automated tractography method for the CRP. To ensure the accurate interpretation of tractographic features, we included individuals with ischemic stroke who were discharged home within three weeks of hospitalization and who regained full independence in daily living, with motor recovery corresponding to Brunnstrom Recovery Stage ≥5 in both affected upper and lower extremities [13]. The Brunnstrom Recovery Stage assesses the motor function of the affected upper (proximal and distal) and lower extremity using a six-point scale ranging from flaccid paralysis to normal function (1 to 6). Stroke diagnosis was confirmed by diffusion-weighted imaging obtained upon arrival, as described previously [14].

All patients received standard acute stroke care in accordance with the Japanese Guidelines for the Management of Stroke 2021 [15]. Throughout their hospitalization, they underwent comprehensive physical, occupational, and speech therapy, with a combined daily total of up to 180 minutes. Informed consent was obtained via an opt-out procedure, and the study was approved by the Institutional Review Board of Hyogo Medical University (Approval No. 4666).

DTI acquisition

As in our previous work [9,10,12], DTI was performed during the second week of admission. Imaging was conducted on a 3.0-T MRI system (MAGNETOM Trio; Siemens, Erlangen, Germany) with a 32-channel head coil. Diffusion-weighted data were acquired using a single-shot echo-planar imaging sequence with diffusion encoding along 30 non-collinear directions (b = 1500 s/mm^2^) and a single non-diffusion-weighted volume (b = 0 s/mm^2^). Eighty contiguous axial slices were obtained with a field of view of 256 × 256 mm, a matrix of 128 × 128, and a slice thickness of 2 mm. Additional acquisition parameters were: echo time, 96 ms; repetition time, 10,900 ms; flip angle, 90°. To correct for eddy-current and susceptibility-induced distortions, additional b = 0 s/mm^2^ images were acquired with opposing phase-encoding directions. High-resolution anatomical images were also obtained using a 3D T1-weighted fast gradient-echo sequence (176 sagittal slices; field of view, 256 × 256 mm; matrix, 256 × 256; slice thickness, 1 mm; echo time, 2.52 ms; repetition time, 1,900 ms; flip angle, 10°).

Image processing

Image preprocessing followed previously described procedures [9,10,12]. Briefly, preprocessing included Gibbs ringing removal, correction of eddy-current and echo-planar imaging-related distortions, and bias-field correction using MRtrix3 [16] and FSL (FMRIB Software Library) [17]. Brain masks were generated from bias-corrected images and applied to all subsequent analyses. Further methodological details, including parameter specifications, are available in the cited literature [9,10,12].

**Figure 1:**
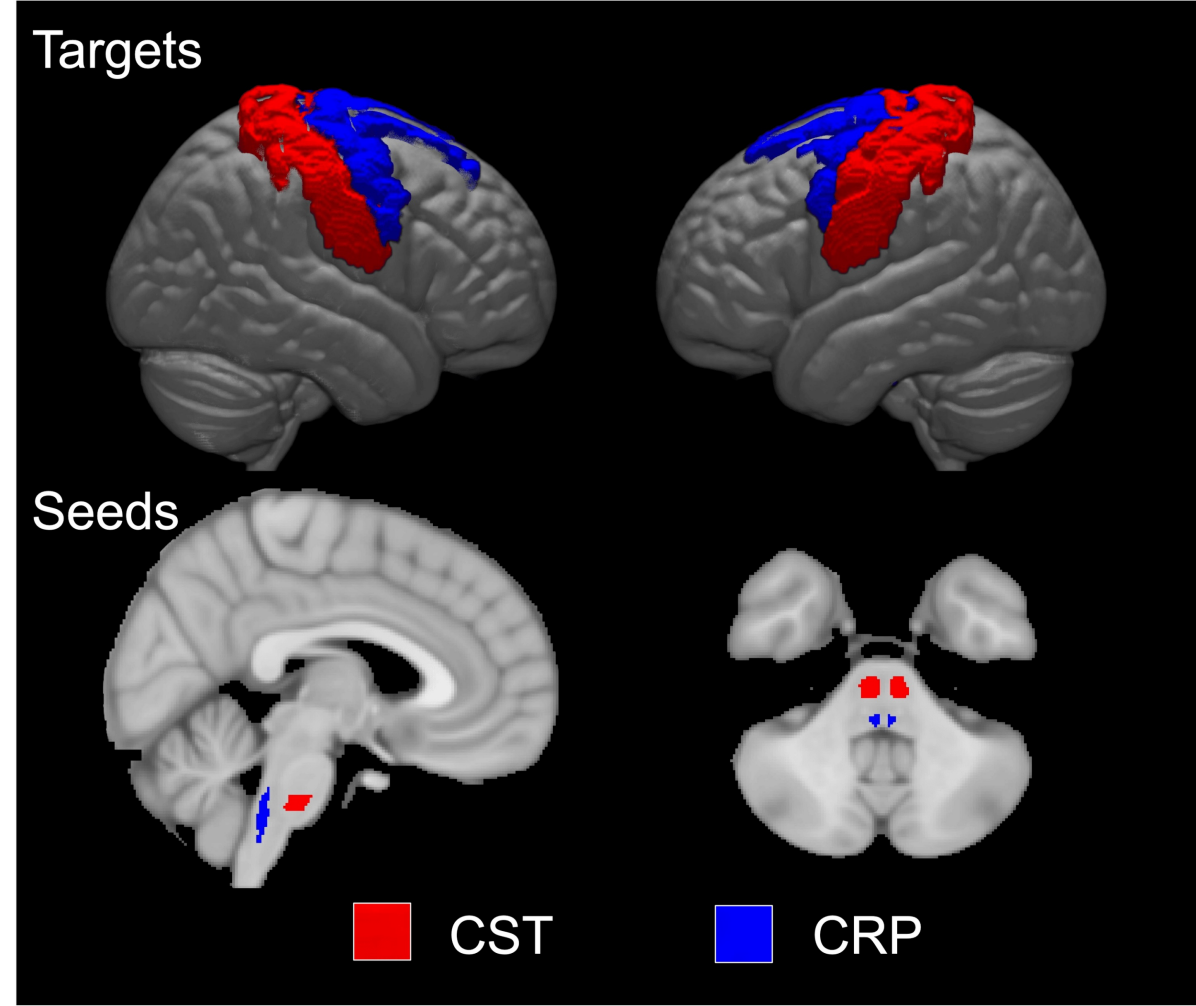
Seed and target regions for the CST and CRP. CRP, corticoreticular pathway; CST, corticospinal tract.

Tractography was performed using the automated framework implemented in FSL. We focused on reconstructing the CST and CRP. CST tractography followed the XTRACT protocol, including the predefined seed, target, and exclusion masks, as well as the specified number of streamlines. For CRP reconstruction, the seed mask was placed in the gigantocellular reticular nucleus based on prior anatomical descriptions [18], and the target mask encompassed the premotor cortex defined in the Juelich histological atlas (index no. 91 for the left hemisphere and no. 92 for the right hemisphere) [19]. Because fractional anisotropy (FA) quantification is sensitive to the chosen threshold, we utilized a cutoff value of 0.01, a standard practice established in earlier research [9,10,12]. An overview of these masks is provided in Figure 1. All remaining tractography parameters were identical to those used for CST reconstruction. For each tract, we calculated tract volume and mean FA. Because partial CST-CRP overlap has been reported [18], the overlapping volume was also quantified. All neuroimaging analyses were performed on a build-to-order desktop workstation (POWERSTEP Tower for Lin4Neuro; Amulet Inc., Tokyo, Japan) running the Lin4Neuro neuroimaging analysis package [20].

Statistical analysis

Given that the present cohort comprised patients with minimal neurological impairment, we compared tract metrics between lesioned and non-lesioned hemispheres using the Wilcoxon signed-rank test (JMP version 19; SAS Institute, Cary, NC, USA). A P-value of < 0.05 was considered statistically significant.

## Results

Table 1 summarizes the cohort of the present study. A total of 15 patients (11 males and four females) with a mean age of 68.7 years (range: 59-83 years) were included. All patients experienced a first-ever unilateral ischemic stroke confined to the corona radiata, with seven cases in the left hemisphere and eight in the right hemisphere.

**Table 1:** Patient characteristics. BRS scores are sequenced as proximal upper extremity-distal upper extremity-lower extremity. BRS, Brunnstrom Recovery Stage; LOS, length of hospital stay.

No.	Age (years)	Sex	Lesioned hemisphere	Lesion site	LOS (days)	BRS
1	83	Male	Left	Corona radiata	17	6-5-6
2	68	Female	Right	Corona radiata	10	6-5-6
3	63	Male	Left	Corona radiata	18	6-6-6
4	61	Male	Left	Corona radiata	11	6-6-6
5	69	Male	Right	Corona radiata	13	5-5-5
6	69	Male	Right	Corona radiata	12	6-6-6
7	59	Male	Right	Corona radiata	15	6-6-6
8	62	Male	Right	Corona radiata	12	6-6-6
9	69	Female	Left	Corona radiata	16	6-5-6
10	72	Male	Right	Corona radiata	12	6-6-6
11	60	Male	Right	Corona radiata	11	6-6-6
12	73	Female	Left	Corona radiata	9	5-5-5
13	74	Male	Left	Corona radiata	16	6-6-6
14	74	Male	Left	Corona radiata	20	6-5-6
15	74	Female	Right	Corona radiata	16	6-6-6

**Figure 2:**
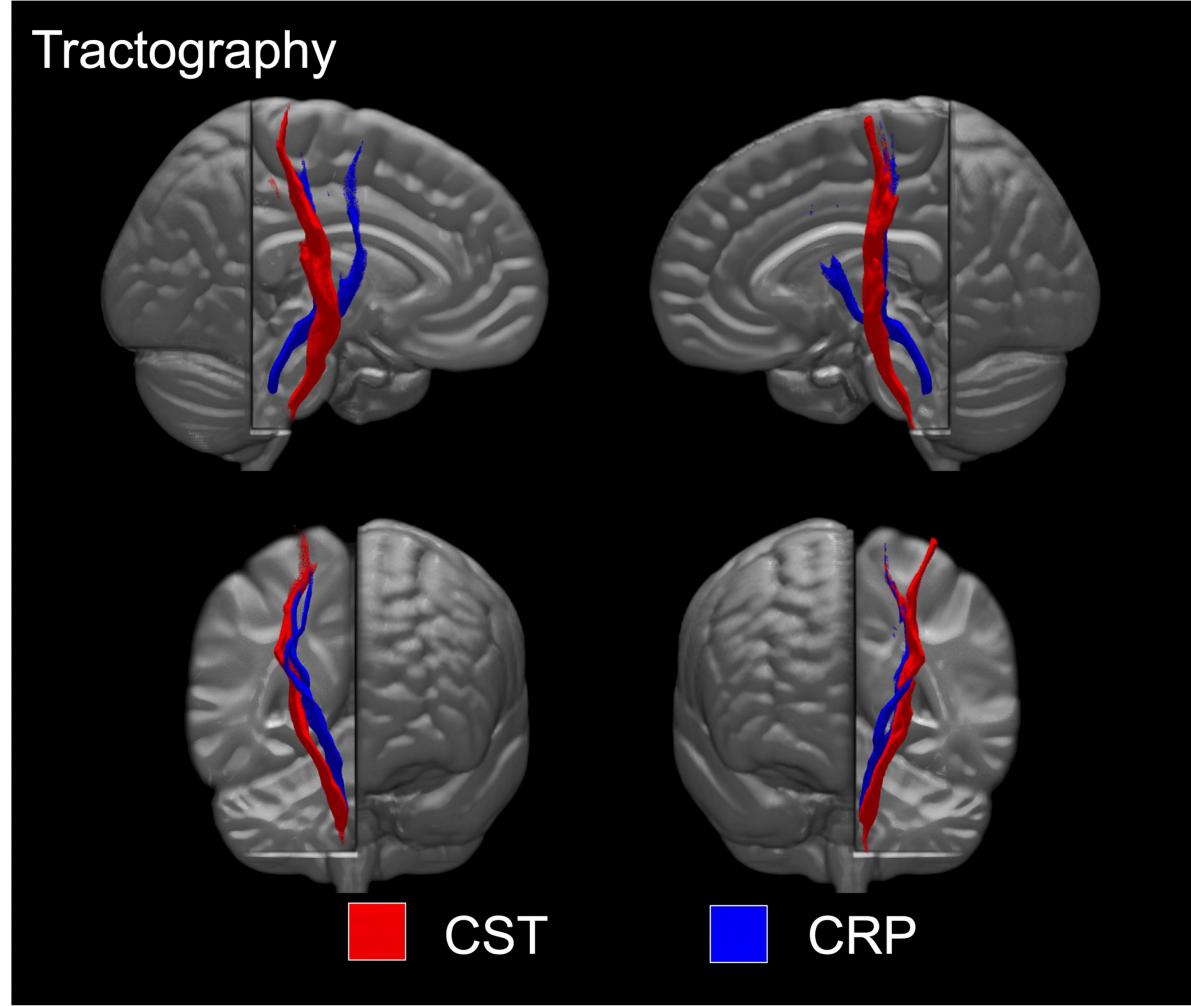
Tractography from an example case. An example (Patient 15 in Tables 1, 2) of the CST and CRP reconstructions. CRP, corticoreticular pathway; CST, corticospinal tract.

Table 2 summarizes the tractography results for all 15 participants, and Figure 2 presents representative CST and CRP reconstructions from patient 15. The automated pipeline successfully reconstructed both tracts in all participants.

**Table 2:** Results obtained from tractography. CRP, corticoreticular pathway; CST, corticospinal tract; FA, fractional anisotropy.

Hemisphere	Lesioned hemisphere					Non-lesioned hemisphere				
Metric	Volume (mL)			FA		Volume (mL)			FA	
No.	CST	CRP	Overlap	CST	CRP	CST	CRP	Overlap	CST	CRP
1	2.50	3.01	1.62	0.544	0.514	2.50	3.14	1.45	0.545	0.566
2	2.55	2.58	0.98	0.545	0.532	2.80	2.54	1.19	0.567	0.544
3	2.85	2.52	1.03	0.567	0.529	2.82	2.77	1.36	0.589	0.556
4	2.66	3.09	0.85	0.593	0.522	2.50	2.94	0.98	0.574	0.589
5	3.06	2.39	1.29	0.476	0.555	2.05	1.78	0.79	0.594	0.589
6	2.63	2.59	0.19	0.470	0.490	2.87	3.03	1.79	0.506	0.501
7	2.52	2.79	0.68	0.576	0.551	3.10	2.94	1.78	0.590	0.569
8	2.70	2.82	0.33	0.587	0.539	2.81	2.91	1.97	0.600	0.588
9	2.09	2.26	0.66	0.504	0.480	2.32	1.95	0.49	0.572	0.518
10	2.59	2.30	0.61	0.559	0.510	2.09	2.50	0.65	0.601	0.507
11	2.34	3.06	0.60	0.491	0.429	1.88	2.52	0.42	0.478	0.485
12	2.11	3.21	0.14	0.496	0.445	2.43	2.18	0.87	0.497	0.517
13	2.65	3.09	1.58	0.537	0.513	1.95	2.15	0.35	0.539	0.542
14	3.43	3.57	1.30	0.556	0.517	3.40	3.44	0.98	0.631	0.573
15	2.68	2.23	0.26	0.580	0.587	2.80	2.24	0.68	0.596	0.566

In the lesioned hemisphere, CST volumes ranged from 2.09 to 3.43 mL (median, 2.63 mL), while CRP volumes ranged from 2.23 to 3.57 mL (median, 2.79 mL). Although the CRP volume generally exceeded the CST volume, the interindividual variability was substantial. Overlapping volumes varied even more widely (0.14-1.62 mL; median, 0.68 mL), indicating heterogeneous spatial proximity between the two pathways. No statistically significant volumetric differences were identified between lesioned and non-lesioned hemispheres.

Microstructural metrics also indicated relatively preserved tract integrity. In the lesioned hemisphere, CST FA values ranged from 0.470 to 0.593 (median, 0.545), whereas CRP FA values ranged from 0.429 to 0.587 (median, 0.517). In contrast, FA values in the non-lesioned CST ranged from 0.478 to 0.631 (median, 0.574), while non-lesioned CRP values ranged from 0.485 to 0.589 (median, 0.556). Group comparisons detected statistically significant differences in FA for both the CST (S = 48, P = 0.004) and the CRP (S = 54, P = 0.001). Thus, although the FA values appeared relatively preserved, subtle microstructural alterations attributable to stroke were evident.

## Discussion

This study demonstrated that automated tractography settings can be adapted to reliably reconstruct the CRP in a manner comparable to the CST. Because the CRP is not included in the original XTRACT protocol [11], we incorporated seed and target regions based on established anatomical literature [18] and histological atlases [19]. The resulting CRP streamlines were positioned medio-anteriorly to the CST, consistent with previous reports [18,21,22]. Furthermore, FA values in the lesioned hemisphere corresponded to the clinical presentation of very mild hemiparesis. Together, these qualitative and quantitative findings support the robustness of the automated tractography pipeline for CRP assessment.

Neuroimaging studies investigating post-stroke motor recovery have increasingly focused on the CRP [3-7]. However, reproducible automated analytical pipelines for CRP evaluation have been lacking. XTRACT was originally developed for research applications and is widely used in research. To facilitate clinical translation, our group previously demonstrated its clinical utility in evaluating extremity function, aphasia, and outcome prediction [9,23,24]. In the present study, we further extended its use by modifying only the seed and target masks, thereby enabling CRP reconstruction with minimal deviation from the established protocol. Given the increasing interest in the contribution of the CRP to motor recovery, this methodology may serve as a useful tool in future rehabilitation research.

The anatomical definition of the CRP varies widely. Some studies have described the CRP as bilateral projections connecting widespread frontal and somatosensory areas to the gigantocellular reticular nuclei [18], whereas others have restricted its definition to ipsilateral projections linking the premotor cortex with the medullary reticular formation and midbrain tegmentum [21]. Here, we adopted a conservative unilateral definition to simplify interpretation. Nevertheless, additional regions, such as Brodmann area 44, which plays a major role in the motor aspects of speech, may contribute to upper-limb motor representations and could be considered potential target regions for future CRP tractography studies [25,26].

We selected patients with mild stroke and minimal residual neurological deficits. Although we initially planned to recruit age-matched healthy controls, this was not feasible in a community hospital setting. While our study focused on patients with mild stroke, it should be noted that lesion severity may affect the reliability of CRP tractography due to potential anatomical distortion or severe Wallerian degeneration. Therefore, further investigation is warranted to determine the robustness of this methodological approach in patients with moderate to severe neurological deficits. However, our approach proved valid: CRP reconstruction was consistently successful in the lesioned hemisphere, and FA values were slightly, but significantly, lower than those in the non-lesioned hemisphere, consistent with very mild clinical deficits.

This study has several limitations. First, FA measurements inherently depend on threshold selection. We adopted a cutoff of 0.01, consistent with previous work [9,23,24], but no universally accepted standard has been established. Different thresholds may produce slightly different results. Second, the cohort consisted exclusively of individuals with first-ever unilateral supratentorial ischemic stroke. Although this homogeneity facilitated interpretation, it may limit generalizability to other lesion types, including infratentorial strokes. Third, the sample size was relatively small (n = 15), reflecting the single-center, retrospective design and the limited number of eligible cases. Finally, our study lacks external validation against manual tractography or expert anatomical assessment. Although manual reconstruction is frequently used as a benchmark, it is highly prone to operator bias and remains non-standardized for the CRP, especially in the context of stroke-induced anatomical distortion. Our automated framework serves as a reproducible alternative that circumvents these subjective limitations. Future research should prioritize multi-center validation and comparisons with consensus-based manual delineations to further strengthen the clinical validity of this automated technique. Nonetheless, CRP reconstruction and quantitative evaluation were consistently achievable across all cases, supporting the feasibility of this method in routine clinical environments. Despite these limitations, we believe that the automated CRP tractography approach presented here provides a practical and clinically applicable framework for future studies and potential integration into stroke rehabilitation practice.

## Conclusions

This study demonstrated the feasibility of incorporating CRP reconstruction into an automated tractography framework by integrating anatomically informed seed and target masks into the existing XTRACT pipeline. The modified workflow reliably reproduced CRP streamlines across all participants and yielded quantitative indices that were consistent with the mild clinical symptoms in our sample. These findings indicate that automated tractography can capture subtle microstructural alterations in both the CST and CRP, even in patients with minimal neurological deficits. By reducing operator dependence and enhancing reproducibility, this approach has the potential to facilitate a more standardized assessment of motor-related white matter pathways in stroke rehabilitation. Although further validation in larger cohorts and across a broader spectrum of stroke severity is required, the present methodology provides a practical foundation for future clinical and research applications involving the CRP.
